# Nonlinear associations of bone age and basal luteinizing hormone with uterine volume in girls with central precocious puberty: a segmented regression analysis

**DOI:** 10.3389/fendo.2026.1952775

**Published:** 2026-10-28

**Authors:** Xin Yuan, Chunyan Cai, Ying Zhang, Wenyong Wu, Ruimin Chen

**Affiliations:** Department of Pediatric Endocrinology, Genetics and Metabolism, Fuzhou First General Hospital Affiliated with Fujian Medical University, Fuzhou Children's Hospital of Fujian Medical University, Fuzhou, China

**Keywords:** basal luteinizing hormone, bone age, central precocious puberty, nonlinear analysis, piecewise regression, uterine volume

## Abstract

**Background:**

The relationships between basal luteinizing hormone (LH), bone age (BA), and uterine volume in girls with precocious puberty are conventionally treated as linear in clinical practice. Whether these associations exhibit nonlinear, threshold-dependent patterns has not been formally investigated.

**Methods:**

This retrospective cross-sectional study included 1, 617 girls with breast development before 8 years of age, of whom 1, 008 had central precocious puberty (CPP) and 609 had premature thelarche (PT). Restricted cubic spline regression was used to test for nonlinearity in the associations of BA and ln-transformed basal LH with ln-transformed uterine volume. Where nonlinearity was confirmed, piecewise linear regression identified breakpoints at which uterine growth accelerated. CPP girls were then cross-classified by the identified thresholds to evaluate their complementary value.

**Results:**

CPP girls had significantly greater BA advancement than PT girls (1.87 ± 1.11 vs. 1.58 ± 1.02 years, P < 0.001). In CPP girls, both BA and basal LH exhibited significant nonlinear associations with uterine volume (both P < 0.0001). A BA breakpoint was identified at 10.6 years: below this threshold, each additional year was associated with a 0.067-unit increase in ln(uterine volume); above 10.6 years, the slope increased to 0.550 (8.2-fold acceleration, P < 0.001). A basal LH breakpoint was identified at 0.67 IU/L, below which the slope was 0.107 per ln-unit and above which it rose to 0.421 (3.9-fold acceleration, P < 0.001). In PT girls, no clinically meaningful breakpoints were found. When CPP girls were stratified by both thresholds, the “Both High” subgroup (16.5%) had a median uterine volume of 2.76 mL (47.6% exceeding 3.0 mL), compared with 1.22 mL (1.4%) in the “Both Low” subgroup (58.6%).

**Conclusions:**

The associations of BA and basal LH with uterine volume in CPP are distinctly nonlinear, with identifiable thresholds beyond which uterine growth accelerates substantially. These breakpoints are proposed as quantitative descriptors of uterine maturation tempo rather than as diagnostic cutoffs; they have high specificity but limited sensitivity, and girls with values below the thresholds require clinical follow-up. These two indicators provide complementary information for characterizing the tempo of uterine maturation.

## Introduction

Central precocious puberty (CPP) is one of the most common referrals to pediatric endocrinology clinics worldwide (1, 2). Recent epidemiological data indicate a rising incidence, further accentuated during the COVID-19 pandemic (3, 4). The condition carries significant clinical consequences, including compromised adult height due to accelerated epiphyseal closure, psychosocial distress, and potentially adverse long-term cardiometabolic outcomes (5, 6). Understanding the tempo of hypothalamic-pituitary-gonadal (HPG) axis activation and its downstream effects on target organ maturation is therefore of considerable clinical importance (7).

Among the downstream effects of estrogen exposure in CPP, uterine maturation and skeletal advancement are two readily measurable phenotypes. Pelvic ultrasound provides a noninvasive window into HPG axis activity by directly visualizing the uterine response to endogenous estrogen stimulation (8, 9). In CPP, uterine dimensions increase in proportion to the duration and intensity of axis activation, making uterine volume a biologically meaningful marker of pubertal progression. Bone age (BA), another key indicator of estrogen exposure, reflects the cumulative effect of sex steroids on skeletal maturation. Chu et al. (10) recently established BA-based reference ranges for uterine volume, highlighting the developmental coupling between skeletal maturation and uterine growth. However, the precise functional form of these relationships—particularly whether they are linear or characterized by threshold effects—has not been formally investigated.

Basal luteinizing hormone (LH), measured with sensitive immunoassays, provides a readily available biochemical indicator of HPG axis activity (11–13). While the diagnostic utility of basal LH for distinguishing CPP from self-limited variants has been extensively studied (14), its quantitative, continuous relationship with uterine maturation has not been characterized using nonlinear modeling approaches. From a physiological perspective, the HPG axis exhibits pulsatile, nonlinear dynamics, suggesting that the gonadotropin–target organ dose–response relationship may not conform to a simple linear model. The identification of thresholds at which uterine growth accelerates could provide valuable information for characterizing the tempo of pubertal progression. Segmented regression analysis is a well-established statistical technique for detecting such inflection points in biomedical data (15), yet it has rarely been applied to pediatric endocrine research. Furthermore, it remains unclear whether these relationships differ between CPP and premature thelarche (PT), a self-limited condition without sustained HPG axis activation.

This study is part of a series of analyses from the same cohort (2018–2024), each addressing a distinct research question. Prior publications examined BMI effects on basal LH (16) and weight-stratified diagnostic cutoffs for CPP (17); another manuscript under review evaluates skeletal risk in incomplete CPP. The present study differs in its research question (nonlinear bone age/LH–uterine volume relationships), its target-organ outcome (uterus), and its analytical approach (restricted cubic spline and piecewise regression). Accordingly, the present study aimed to: (1) characterize the shape of the associations between BA, basal LH, and uterine volume in a large cohort of girls with precocious pubertal development; (2) identify breakpoints at which uterine growth accelerates using piecewise linear regression; and (3) evaluate whether combining BA and basal LH thresholds provides complementary information for characterizing the tempo of uterine maturation.

## Methods

### Study population

This was a retrospective cross-sectional study conducted at a single center. Girls who were admitted to our center for early breast development between January 2018 and April 2024 were included. All data for each girl, including anthropometric measurements, BA, basal LH, GnRH stimulation test results, and pelvic ultrasound, were collected during a single initial visit. Inclusion criteria were: (1) breast development before 8 years of age; (2) complete data on BA, basal LH, GnRH stimulation test results, and pelvic ultrasound. Participants were classified into two groups according to GnRH stimulation test results: CPP (peak LH ≥ 5.0 IU/L) and PT (peak LH < 5.0 IU/L). Exclusion criteria were peripheral precocious puberty, intracranial lesions, congenital adrenal hyperplasia, hypothyroidism, and prior GnRH agonist treatment. The study was approved by the Medical Ethics Committee of Fuzhou Children’s Hospital of Fujian Medical University (Approval No. 201927), and was carried out in compliance with the Declaration of Helsinki.

### Data collection

Chronological age, height, and weight were recorded at the initial visit. Body mass index standard deviation score (BMI SDS) was calculated using age- and sex-specific national reference data. BA was assessed by a single experienced pediatric endocrinologist using the Tanner-Whitehouse 3 (TW3) method. Basal LH was measured from a single fasting blood sample by chemiluminescence (Siemens Healthcare Diagnostics, Los Angeles, CA, USA). Uterine volume was calculated from transabdominal ultrasound measurements using the formula 0.523 × longitudinal × anteroposterior × transverse diameter, performed by a single experienced pediatric sonographer.

### Statistical analysis

Continuous variables were expressed as mean ± standard deviation or median (interquartile range) depending on normality. Between-group comparisons were performed using Student’s t-test or Mann-Whitney U test as appropriate.

Given the right-skewed distributions of basal LH and uterine volume, natural logarithmic transformation was applied to both variables. Because CPP and PT represent etiologically distinct conditions with fundamentally different HPG axis dynamics, all analyses were performed separately by group rather than in a pooled model. Within each group, Pearson correlation and partial correlation coefficients (adjusted for chronological age and BMI SDS) were calculated between BA, ln(LH), and ln(UV).

Restricted cubic spline regression with three knots (placed at the 10th, 50th, and 90th percentiles of the predictor distribution) was used to test for nonlinearity in the relationships of BA and ln(LH) with ln(UV) within each group, adjusting for chronological age and BMI SDS. Where significant nonlinearity was confirmed, piecewise linear regression was performed to identify breakpoints (18). Candidate breakpoints were evaluated across biologically plausible ranges (BA: 9.0-11.0 years, step 0.2; ln[LH]: -0.5 to 0.5, step 0.1). At each candidate threshold, a two-segment linear model was fitted, allowing distinct regression slopes on either side of the candidate breakpoint, adjusting for chronological age and BMI SDS. The optimal breakpoint was selected as the value maximizing adjusted R² with the constraint of a significantly positive slope change (P < 0.05).

To evaluate the complementary value of the two indicators, CPP girls were cross-classified according to the identified breakpoints (BA > 10.6 years; basal LH > 0.67 IU/L), and uterine volume was compared across the resulting subgroups using the Kruskal-Wallis test.

Loess-smoothed scatterplots were generated for visualizing the relationships of BA and ln(LH) with ln(UV) in each group. All analyses were performed using R version 4.6.0. A two-sided P < 0.05 was considered statistically significant.

## Results

### Baseline characteristics

A total of 1, 617 girls with breast development before 8 years of age were included, comprising 1, 008 (62.3%) with CPP and 609 (37.7%) with PT. Baseline characteristics stratified by group are presented in **Table 1**. Girls with CPP were slightly older at presentation than those with PT (P < 0.001). The CPP group exhibited more advanced BA and greater BA advancement (BA − CA), higher basal LH levels, and larger uterine volume (all P < 0.001). BMI SDS did not differ between groups (0.49 ± 1.19 vs. 0.51 ± 1.27, P = 0.672).

**Table 1:** Baseline characteristics of the study population.

Characteristic	PT	CPP	P value
n	609	1008	/
Age, years	7.20 ± 0.87	7.57 ± 0.92	<0.001
BMI SDS	0.51 ± 1.27	0.49 ± 1.19	0.672
BA, years	8.78 ± 1.48	9.61 ± 1.60	<0.001
BA advancement, years	1.58 ± 1.02	1.87 ± 1.11	<0.001
Basal LH, IU/L	0.12 [0.10–0.18]	0.29 [0.16–0.82]	<0.001
Uterine volume, mL	1.11 [0.91–1.38]	1.42 [1.09–1.90]	<0.001

Data are presented as mean ± SD for normally distributed variables and median [interquartile range] for non-normally distributed variables. P values from independent-samples t-test for normal variables and Mann–Whitney U test for non-normal variables.

Given the right-skewed distributions of basal LH and uterine volume, natural logarithmic transformation was applied to approximate normality.

### Associations between BA, basal LH, and uterine volume

Stratified Pearson correlation and partial correlation coefficients (adjusted for chronological age and BMI SDS) are presented in **Table 2**. In the CPP group, BA (crude r = 0.531, adjusted r = 0.305, both P < 0.001) and ln(LH) (crude r = 0.583, adjusted r = 0.490, both P < 0.001) were positively correlated with ln(UV). In the PT group, corresponding correlations were weaker: BA (crude r = 0.308, adjusted r = 0.236, both P < 0.001) and ln(LH) (crude r = 0.345, adjusted r = 0.316, both P < 0.001).

**Table 2:** Stratified correlation analysis between predictors and ln(uterine volume).

Group	n	Variable pair	Crude r	Crude P	Adjusted r*	Adjusted P
CPP	1008	BA – ln(UV)	0.531	<0.001	0.305	<0.001
		ln(LH) – ln(UV)	0.583	<0.001	0.490	<0.001
PT	609	BA – ln(UV)	0.308	<0.001	0.236	<0.001
		ln(LH) – ln(UV)	0.345	<0.001	0.316	<0.001

BA, bone age; ln(LH), natural logarithm of basal luteinizing hormone; ln(UV), natural logarithm of uterine volume. *Partial correlation coefficients adjusted for chronological age and BMI SDS.

### Identification of critical breakpoints in CPP

Restricted cubic spline regression confirmed nonlinear relationships in the CPP group for both BA (nonlinear F = 111.01, P < 0.0001) and ln(LH) (nonlinear F = 41.41, P < 0.0001). In the PT group, nonlinearity was weaker (BA: F = 15.17, P = 0.0001; ln[LH]: F = 7.05, P = 0.008).

Piecewise linear regression, adjusted for chronological age and BMI SDS, was used to identify breakpoints at which uterine growth accelerated. Results are summarized in **Table 3**, and the corresponding Loess-smoothed trajectories are shown in **Figures 1**, **2**.

**Table 3:** Segmented regression analysis: optimal breakpoints for uterine growth acceleration in CPP girls.

Predictor	Breakpoint	Uterine growth pattern before breakpoint	Uterine growth pattern after breakpoint	Acceleration factor	P
Bone age	10.6 yr	Gradual	8.2× faster	8.2×	<0.001
Basal LH	0.67 IU/L	Gradual	3.9× faster	3.9×	<0.001

All models adjusted for chronological age and BMI SDS. In PT girls, no significant breakpoints were identified for either indicator.

**Figure 1:**
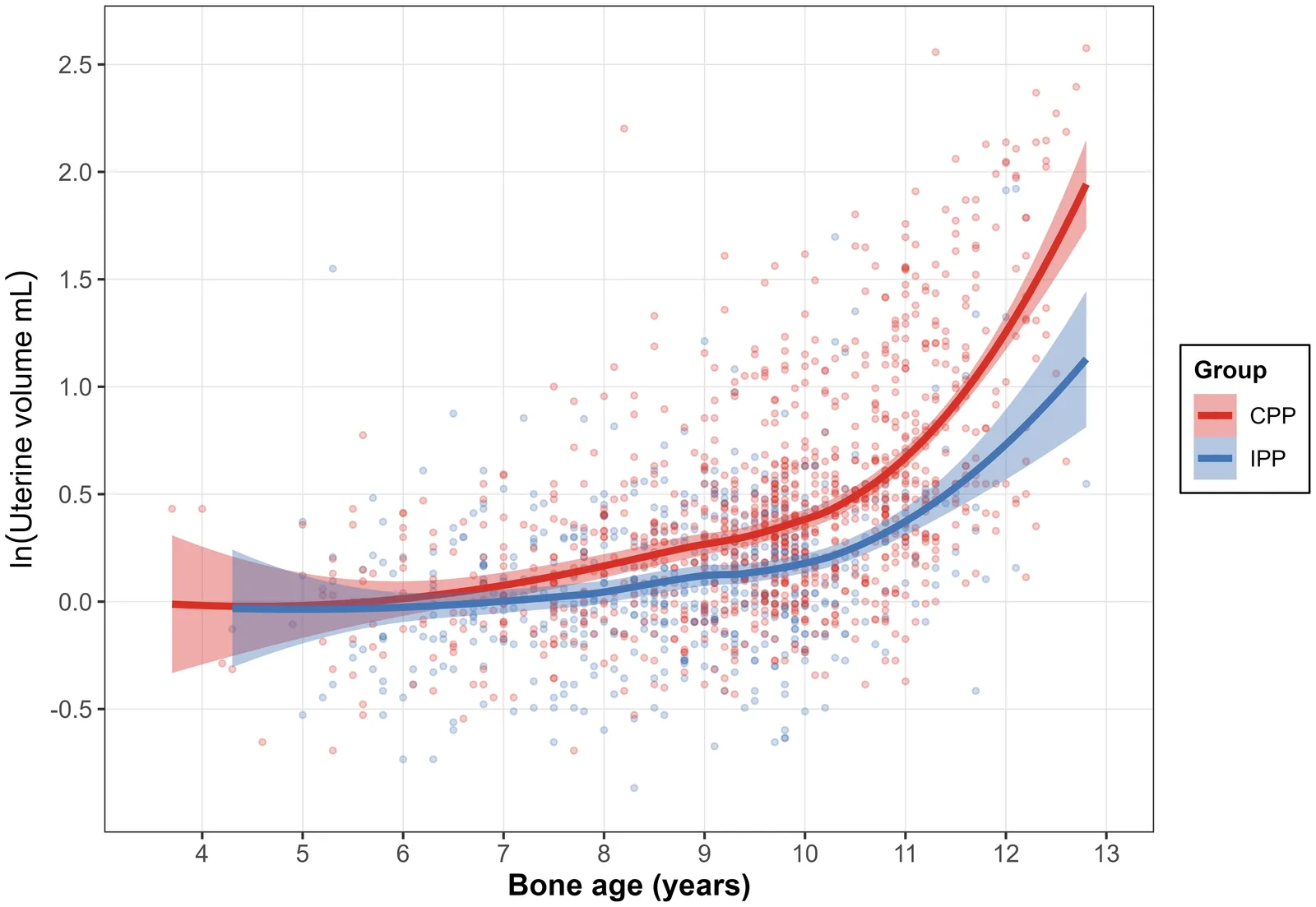
Loess-smoothed scatterplot of bone age versus ln(uterine volume) stratified by group. CPP, central precocious puberty (red); PT, premature thelarche (blue). Shaded areas represent 95% confidence bands. The CPP curve demonstrates a distinct upward inflection beyond 10.6 years of bone age, whereas the PT curve remains relatively flat.

**Figure 2:**
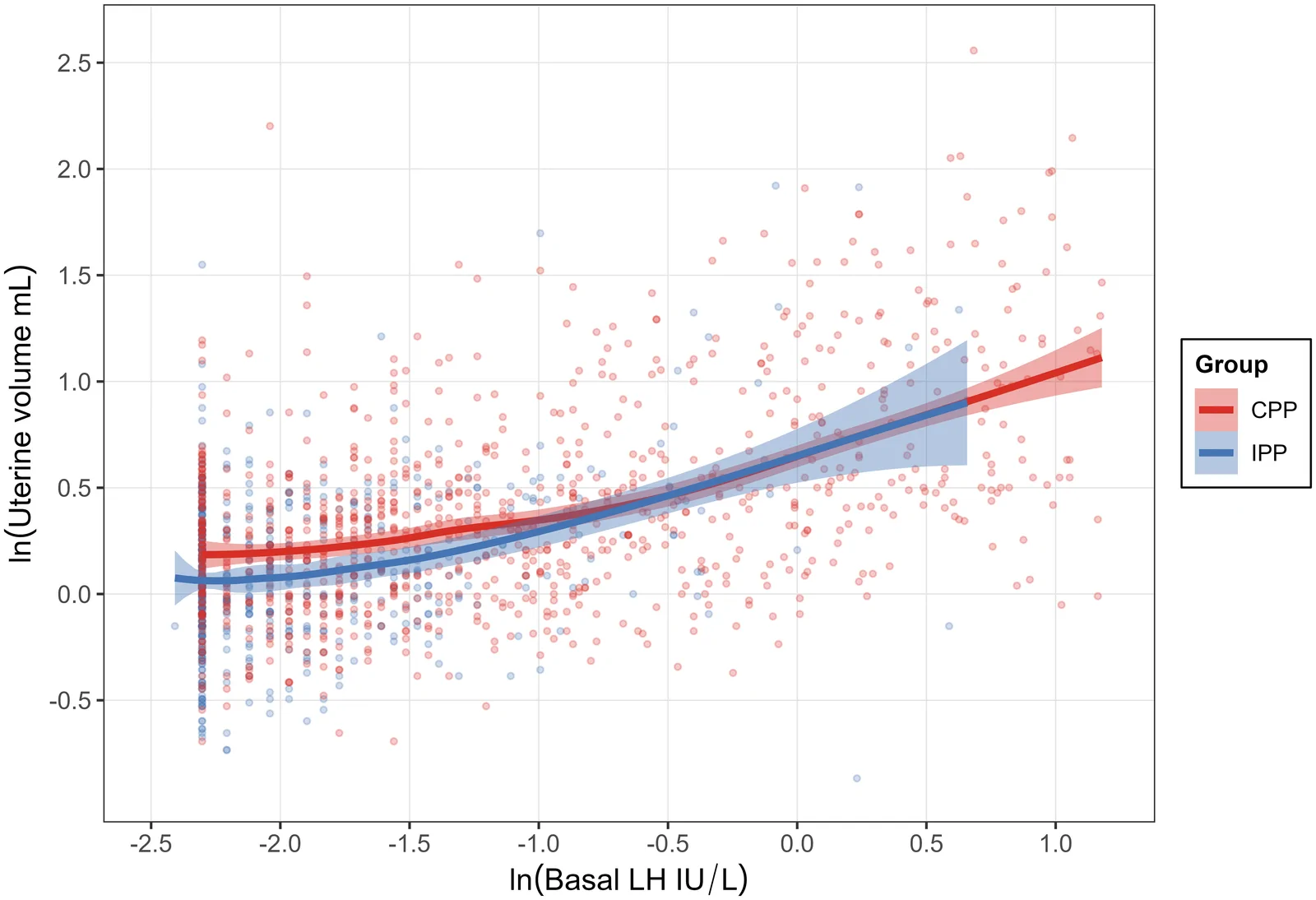
Loess-smoothed scatterplot of ln(basal LH) versus ln(uterine volume) stratified by group. CPP (red) exhibits progressive steepening beyond ln(LH) ≈ −0.4 (raw LH ≈ 0.67 IU/L), while PT (blue) shows minimal increase across the LH range.

For BA, the optimal breakpoint was 10.6 years. Below this threshold, uterine growth was gradual; above 10.6 years, the growth rate accelerated 8.2-fold (P < 0.001). For basal LH, the optimal breakpoint was 0.67 IU/L, below which growth was gradual and above which the rate accelerated 3.9-fold (P < 0.001). Both piecewise models fitted the data significantly better than corresponding linear models.

In the PT group, no significant breakpoints were identified for either BA or basal LH. As shown in **Figures 1**, **2**, uterine growth in PT follows a largely linear trajectory.

### Complementary value of combined assessment

CPP girls were cross-classified according to the identified breakpoints (BA > 10.6 years; LH > 0.67 IU/L) (**Table 4**). In the Both High subgroup (n = 166, 16.5%), median uterine volume was 2.76 mL (IQR: 1.73–4.66) and 47.6% had UV > 3.0 mL. In the Either High subgroup (n = 251, 24.9%), median UV was 1.61 mL (IQR: 1.25–2.34) with 14.3% exceeding 3.0 mL. In the Both Low subgroup (n = 591, 58.6%), median UV was 1.22 mL (IQR: 0.98–1.51) with 1.4% exceeding 3.0 mL. Uterine volume differed significantly across the three strata (P < 0.0001) (**Figure 3**).

**Table 4:** Combined risk stratification using dual thresholds in CPP girls.

Risk stratum	n (%)	Uterine volume (mL)	UV > 3.0 mL (%)
Both High	166 (16.5)	2.76 [1.73–4.66]	47.6
Either High	251 (24.9)	1.61 [1.25–2.34]	14.3
Both Low	591 (58.6)	1.22 [0.98–1.51]	1.4

Both High: bone age > 10.6 years and basal LH > 0.67 IU/L; Either High: one criterion met; Both Low: neither criterion met. Uterine volume expressed as median [interquartile range]. Kruskal-Wallis test P < 0.0001 across the three strata.

**Figure 3:**
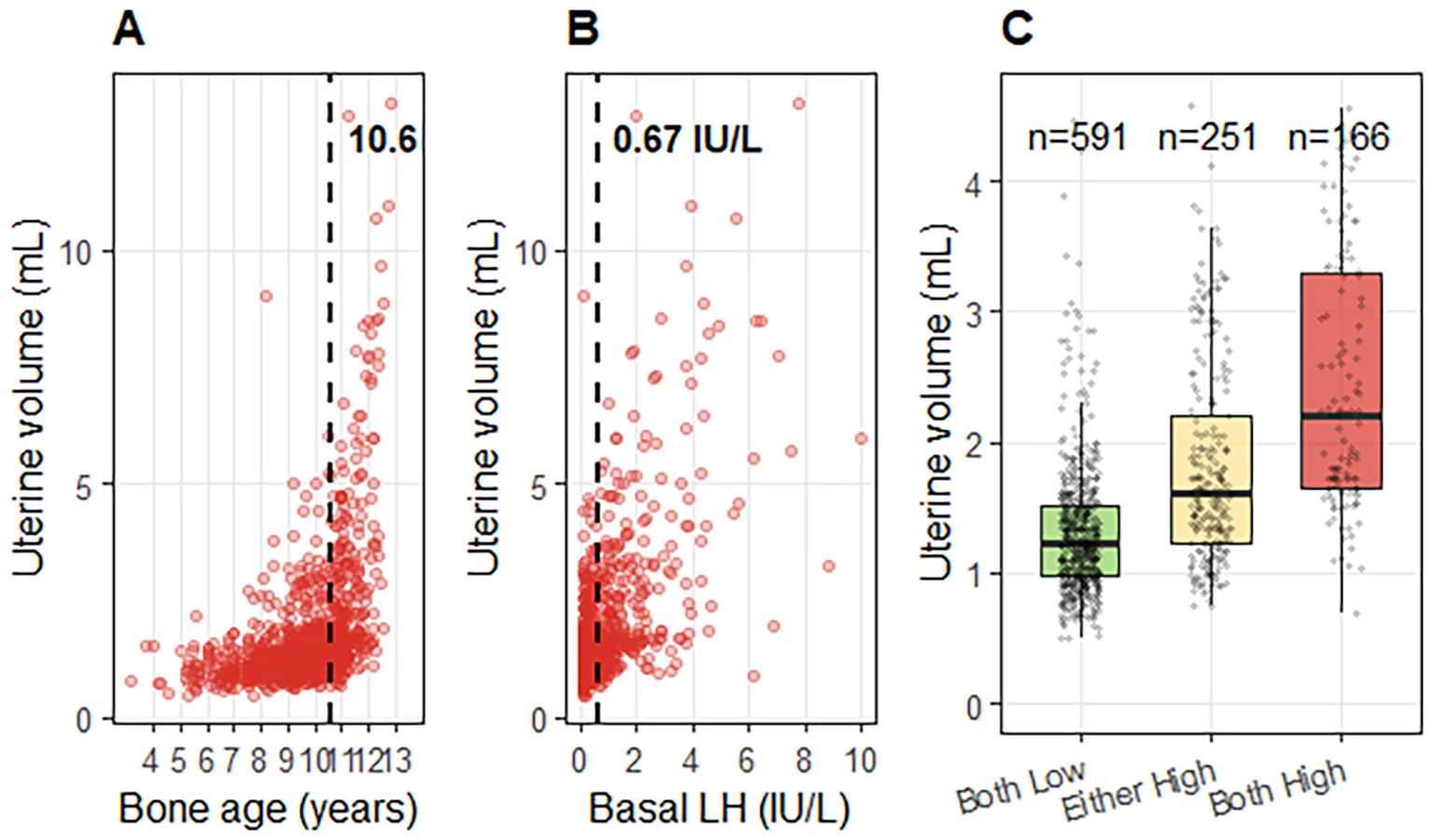
Combined dual-threshold assessment in CPP girls. **(A)** Scatterplot of bone age versus uterine volume with the 10.6-year breakpoint marked (dashed line). **(B)** Scatterplot of basal LH versus uterine volume with the 0.67 IU/L breakpoint marked (dashed line). **(C)** Boxplot comparing uterine volume across the three strata: Both High (n = 166), Either High (n = 251), Both Low (n = 591). Boxes show median and interquartile range; whiskers extend to 1.5× IQR. Kruskal-Wallis test P < 0.0001.

## Discussion

In this large cross-sectional study of 1, 617 girls with precocious pubertal development, we demonstrate-using restricted cubic spline regression combined with piecewise regression-that the associations of basal LH and BA with uterine volume are distinctly nonlinear in CPP but not in PT. To our knowledge, this is the first study to apply nonlinear dose-response modeling to characterize how uterine maturation progresses in relation to these two readily available clinical indicators. Two breakpoints were identified: a BA of 10.6 years and a basal LH of 0.67 IU/L, above which uterine growth accelerates by 8.2-fold and 3.9-fold, respectively. These findings provide novel quantitative insights into the tempo of HPG axis-driven uterine maturation during CPP progression.Importantly, these breakpoints characterize where and how abruptly the rate of uterine growth changes along the bone age and LH continua. They are not intended as diagnostic cutoffs for distinguishing CPP from PT, but rather as quantitative descriptors of the tempo of uterine maturation during CPP progression.

The nonlinear, threshold-type relationship between basal LH and uterine volume is biologically plausible and reflects the pulsatile nature of gonadotropin secretion (19, 20). Below 0.67 IU/L, LH levels likely reflect the low-amplitude secretory pattern of early activation; above this threshold, LH concentrations signify sustained pulsatile secretion capable of driving progressive uterine growth. This interpretation is consistent with the molecular framework of pubertal progression described by Argente et al. (19), in which a cascade of neuroendocrine events transforms the HPG axis from a quiescent to a fully activated state. Notably, Çömlek et al. (9) independently reported that a basal LH cutoff of 0.67 IU/L—identical to our breakpoint—was significantly associated with pubertal GnRH response, providing cross-study corroboration.

The BA breakpoint at 10.6 years highlights the developmental coupling between skeletal maturation and uterine growth. While BA advancement is a well-recognized feature of CPP, its quantitative relationship with uterine dimensions has not been previously characterized using nonlinear approaches. Our finding of an 8.2-fold increase in uterine growth rate beyond BA 10.6 years suggests that this skeletal age marks a transition point in the tempo of peripheral estrogen action. The BA-based reference ranges published by Chu et al. (10) show a similar steepening of the uterine volume-BA curve beyond approximately 10 years in normally developing girls, suggesting that the acceleration we observed in CPP may represent an amplification of a physiological trajectory rather than a qualitatively distinct phenomenon.

The LH–uterine volume and BA–uterine volume relationships differed markedly between CPP and PT. In PT girls, no LH-related breakpoint was identified, and uterine growth followed a largely linear trajectory. This absence of a breakpoint in PT is largely inherent to the definition and natural history of the condition itself: PT is defined as isolated breast development without sustained HPG axis activation, and therefore lacks the progressive gonadotropin drive required to generate threshold-type uterine growth acceleration. Once sustained central activation occurs, girls are reclassified as having entered true puberty rather than remaining in the PT category. The absence of LH-driven growth acceleration in PT thus highlights the specificity of the nonlinear pattern to progressive puberty (6, 21, 22).

The dual-threshold classification scheme provides a practical framework for staging the tempo of uterine maturation in CPP. The three-tier system clearly separated girls by uterine volume, with median values of 2.76 mL, 1.61 mL, and 1.22 mL in the Both High, Either High, and Both Low groups, respectively. These findings indicate that BA and basal LH contribute complementary information: BA reflects cumulative estrogen exposure on both skeletal and uterine tissues, while basal LH provides a snapshot of current central drive. All models were adjusted for BMI SDS, ensuring that the observed associations were independent of adiposity-related confounding.

It should be emphasized that the identified breakpoints are not diagnostic thresholds. From a diagnostic perspective, these thresholds have high specificity but limited sensitivity: girls with values below the breakpoints cannot be considered free of CPP, and clinical follow-up remains necessary to clarify the diagnosis and to differentiate CPP from PT in such cases. The value of these breakpoints lies in characterizing the tempo of uterine maturation among girls with established CPP, rather than in discriminating CPP from PT.

Prior studies have largely focused on diagnostic models distinguishing CPP from PT using binary classification endpoints (8, 9, 23). Our study addresses a different question: characterizing the tempo of uterine maturation once CPP is established. The breakpoints described here may help distinguish faster from slower progression among girls already diagnosed with CPP, complementing rather than competing with existing diagnostic tools.

The strengths of this study include the large sample size (N = 1, 617), among the largest cohorts reported for this clinical question; the rigorous analytical framework combining RCS for nonlinearity detection with piecewise regression for breakpoint identification-an approach not previously applied in this context; and the use of logarithmic transformation to address the marked right skewness of basal LH and uterine volume distributions.

Several limitations should be acknowledged. First, the retrospective, single-center design limits generalizability; external validation in independent cohorts is needed. Second, the lower mean bone age in the PT group (8.78 ± 1.48 years) limits definitive exclusion of a breakpoint beyond 10.6 years in this population; studies including older PT girls or longitudinal follow-up through the transition to CPP are needed to determine at what point the nonlinear pattern emerges. Third, the cross-sectional design precludes causal inference; prospective longitudinal studies are required to confirm that crossing the LH threshold temporally precedes accelerated uterine enlargement. Fourth, basal LH was measured from a single fasting sample, which may not fully capture pulsatile gonadotropin secretion (11). Fifth, estradiol levels were not available, precluding direct assessment of the intermediate hormonal step between LH elevation and uterine growth. Finally, transabdominal ultrasound assessment of uterine volume may be influenced by bladder filling and operator variability, although the use of a single experienced sonographer mitigated this concern.

## Conclusion

In conclusion, this study provides evidence for nonlinear, threshold-dependent relationships between basal LH, BA, and uterine volume in CPP girls, with breakpoints identified at BA 10.6 years and basal LH 0.67 IU/L. Used together, these two readily available clinical indicators capture complementary dimensions of CPP progression and provide a quantitative framework for staging the tempo of uterine maturation. These breakpoints are proposed as quantitative descriptors of uterine maturation tempo in CPP, not as diagnostic criteria. They have high specificity but limited sensitivity, and girls with values below these thresholds require clinical follow-up to clarify the diagnosis. These thresholds require external validation before they can be applied in clinical practice. Future research should focus on external validation of these breakpoints in independent populations and longitudinal studies to establish the temporal dynamics of threshold crossing and uterine growth acceleration, and evaluation of whether progression staging based on these thresholds can inform clinical monitoring.

## Data Availability

The original contributions presented in the study are included in the article/supplementary material. Further inquiries can be directed to the corresponding author.
